# The clinical applications of dual-layer spectral detector CT in digestive system diseases

**DOI:** 10.1007/s00330-024-11290-6

**Published:** 2024-12-19

**Authors:** Yidi Chen, Xijiao Liu, Hanjiang Zeng, Jinge Zhang, Zhengyan Li, Bin Wu, Zixing Huang, Bin Song

**Affiliations:** 1 Depatment of Radiology, West China Hospital, Sichuan University, Chengdu, China; 2 Department of Radiology, The First Affiliated Hospital of Guangxi Medical University, Nanning, China; 3 Department of Radiology, Sanya People’s Hospital, Sanya, China

**Keywords:** Computed tomography, Multidetector computed tomography, Digestive system, Liver, Gastrointestinal tract

## Abstract

**Objective:**

Dual-layer spectral detector CT (DLCT) has several advantages in clinical practice, this study aims to reveal the clinical applications of DLCT in digestive system diseases.

**Materials and methods:**

We searched PubMed and Cochrane Reviews for articles published from January 1, 2010 to May 31, 2024, using the terms “dual-layer spectral detector CT” or “dual-layer CT” combined with “hepatic fat” or “hepatic fibrosis” “hepatocellular carcinoma” or “pancreatic ductal adenocarcinoma” or “pancreatic neuroendocrine tumors” or "gastric cancer" or "colorectal cancer" or "Crohn’s disease" or "bowel ischemia" or "acute abdominal conditions".

**Results:**

DLCT consists of a top layer sensitive to lower-energy photons and a bottom layer sensitive to higher-energy photons. This configuration enables simultaneous acquisition of two energy spectra from a single X-ray beam ensuring consistent spatial alignment and temporal resolution. Spectral raw images allow image post-processing to improve image quality, reduce radiation doses and contrast media doses, and generate multiple quantitative parameters. It has broad potential for early detection, accurate staging, efficacy assessment, and prognosis prediction of liver, pancreatic, and gastrointestinal diseases, as well as for the assessment of digestive system vasculature.

**Conclusions:**

DLCT not only provides valuable information for the clinical diagnosis and therapeutic effect evaluation of digestive system diseases but also may play a more important role in the overall management of digestive diseases and in the decision-making of individualized medicine.

**Key Points:**

***Question*** *What are the advantages of DLCT compared to traditional single-energy CT in the early detection, staging, and therapeutic evaluation of digestive system diseases*?

***Findings*** *DLCT enhances image quality, improves tissue characterization, and allows for multi-parametric analysis, making it superior in detecting and evaluating liver, pancreatic, and gastrointestinal diseases*.

***Clinical relevance*** *DLCT provides high-quality, multi-parametric imaging that improves the accuracy of diagnosing digestive diseases, facilitates more precise treatment planning, and enhances monitoring of treatment response, ultimately contributing to better patient management and prognosis*.

## Introduction

Digestive system diseases pose significant public health concerns worldwide [1]. According to the latest survey released by the National Cancer Center, colorectal cancer (CRC), gastric cancer, and liver cancer rank among the top five most common malignant tumors in China, with liver cancer, gastric cancer, CRC, and esophageal cancer being the 2nd to 5th leading causes of cancer-related deaths [2]. Therefore, early diagnosis, comprehensive evaluation, and timely treatment of digestive system diseases are clinically important.

CT is the primary imaging choice for digestive system diseases [3]. However, traditional single-energy CT can provide limited information for the diagnosis of disease and the assessment of biological characteristics. Dual-layer spectral detector CT (DLCT) is an advanced dual-energy CT technology that uses dual-layer detectors to simultaneously collect low- and high-energy data, achieving “homogeneous, simultaneous, and codirectional” spectral data acquisition [4]. “Homogeneous” refers to the uniform acquisition of spectral data across the entire field of view, without variation in energy separation. “Simultaneous” indicates that both low- and high-energy data are acquired at the same time, eliminating temporal mismatches that could degrade image quality. “Codirectional” means that the spectral data are acquired along the same path, ensuring that the same anatomical structures are imaged at both energy levels, thus increasing the accuracy of post-processed images. DLCT facilitates the collection of precise spectral data, ensuring quantitative accuracy without being affected by variations in patient size or acquisition parameters [5]. Multiple images can be obtained via post-processing, including the generation of virtual monoenergetic images (VMIs), iodine quantification maps, and effective atomic number (*Z*-effective) maps, which are beneficial for the accurate characterization of digestive system pathologies [6].

Although dual-source CT (DSCT) may provide reliable spectral separation, DLCT offers distinct advantages in specific clinical scenarios. These include its ability to retrospectively generate spectral images from a single acquisition without the need for pre-selection of protocols, thus providing flexibility in clinical workflows, Furthermore, DLCT is particularly beneficial in cases where patient-specific factors, such as CT scans for patients with severe liver disease or acute abdomen may complicate the pre-selection of energy levels. The continued innovation lies in the evolving applications of DLCT in digestive system pathologies, particularly in its ability to provide multi-parametric imaging that enhances diagnostic accuracy, therapeutic assessment, and prognostic evaluation.

The aforementioned multiparameter imaging of DLCT can offer valuable reference information for the accurate diagnosis and prognosis of digestive system diseases. This paper reviews the general advantages of DLCT in the liver, pancreas, gastrointestinal tract, and digestive system vasculature.

## The technical details of DLCT

DLCT utilizes a unique detector configuration in which two layers of detectors are stacked on top of each other within the CT gantry. The top layer, or front detector, is designed to be more sensitive to lower-energy photons, whereas the bottom layer, or rear detector, captures higher-energy photons that have passed through the first layer. This configuration allows for the simultaneous acquisition of two distinct energy spectra from a single X-ray beam, ensuring perfect temporal and spatial alignment between the low- and high-energy datasets. The physics of photon interaction within this system relies on the principle that lower-energy photons are more likely to be absorbed by the front detector, while higher-energy photons penetrate through to the rear detector. This separation of energy levels enables the generation of spectral data that can be used for advanced post-processing techniques, such as virtual monoenergetic imaging (VMI), iodine quantification, and material decomposition [6].

The dual-layer approach has several advantages in clinical practice. First, it enhances image quality by reducing beam-hardening artifacts, which are common in conventional single-energy CT and can obscure diagnostic details, particularly in dense tissues such as bone or areas with metal implants [7]. Second, the technology improves dose efficiency, as it eliminates the need for multiple scans or energy-switching protocols, reducing patient radiation exposure while maintaining high image quality. Third, the simultaneous acquisition of spectral data allows for retrospective reconstruction of images, providing flexibility in clinical workflows and enabling the extraction of additional diagnostic information without the need for repeat scanning. These features make DLCT particularly valuable in the imaging of the digestive system [3], where precise tissue characterization [8] and artifact reduction are crucial for accurate diagnosis and treatment planning.

## The clinical application of DLCT in hepatic diseases

### Hepatic fat quantification

The quantification of hepatic fat offers objective results independent of CT values, which are relevant prognostic parameters (e.g., hepatic steatosis or steatohepatitis). Hepatic steatosis could be detected in DLCT scans from routine clinical practice, which provide retrospective spectral information independent of the imaging mode. DLCT overcomes the limitation of fat quantification, as source-based dual-energy CT techniques such as fast kVp-switching CT or DSCT require prior selection of the dual-energy imaging mode. In a recent study [8], researchers developed a material decomposition algorithm for fat quantification in phantoms and validated it in vivo for liver and skeletal muscle using DLCT. The results showed excellent intraclass correlation coefficients in the phantoms at both 120 kV and 140 kV (the DLCT vs MR chemical shift relaxometry was 0.98, and the DLCT vs MR spectroscopy was 0.96). Similarly, Emilie et al [9] revealed that a three-material decomposition algorithm for hepatic fat quantification using dual-layer CT could be used to evaluate liver steatosis with good correlation with MRI.

### Hepatic fibrosis

**Fig. 1:**
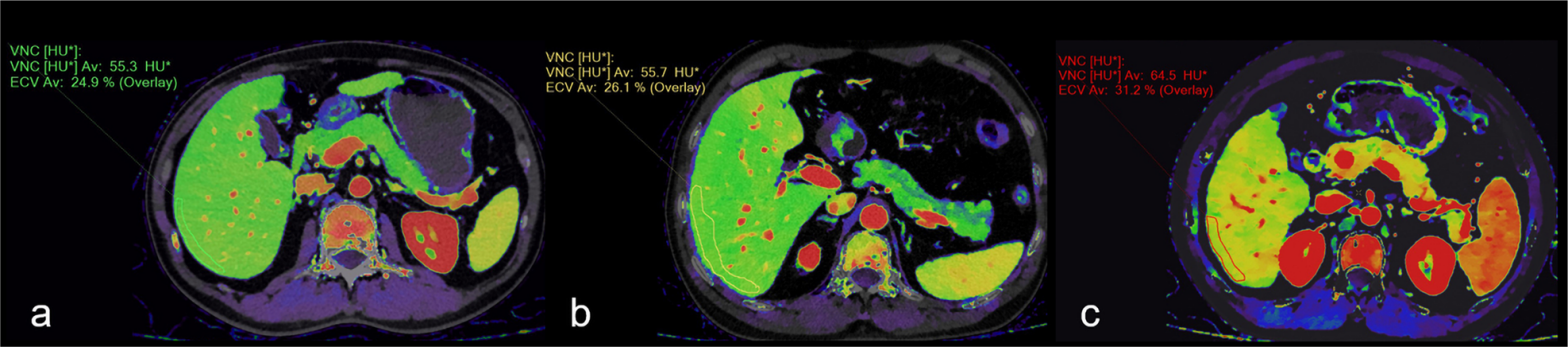
Liver ECV postprocessing of DLCT images in the arterial phase. **a** A 37-year-old male with a normal liver. **b** A 69-year-old male patient with pathologically confirmed liver fibrosis grade S2. **c** A 45-year-old male patient with pathologically confirmed liver fibrosis grade S4. The liver ECV values of the three patients gradually increased to 24.9%, 26.1%, and 31.2%, respectively

In recent years, DLCT has demonstrated relative advantages over traditional single-energy CT in the noninvasive assessment of hepatic fibrosis grade. DLCT requires only one scan to obtain spectral images to calculate the extracellular volume (ECV). The utility of ECV for quantitatively evaluating hepatic fibrosis has been confirmed. Bandula et al [10] reported a correlation between liver ECV measured using conventional CT imaging and the proportion of collagen fibers in biopsy tissue. Morita et al [11] demonstrated a significantly greater ECV of DLCT in hepatic fibrosis stage F4 than in stages F0, F1, F2, and F3 and a positive correlation between the hepatic fibrosis stage and ECV. The aforementioned studies highlight the potential of ECV in evaluating hepatic fibrosis. DLCT enables a more convenient ECV technique, which is expected to play a more significant role in hepatic fibrosis assessment in the future. Figure 1 shows the ECV values of DLCT for normal liver and liver fibrosis patients.

### Hepatocellular carcinoma (HCC)

The application of DLCT in HCC primarily focuses on early tumor detection and the assessment of biological behaviors. Compared with conventional CT images, low keV VMIs with DLCT can improve the quality of abdominal images and increase the contrast between lesions and normal liver parenchyma, thereby improving the detection rate of lesions [12, 13]. Contrast-enhanced DLCT also increases the confidence of radiologists in depicting lesion characterization [14]. A previous study [15] confirmed that VMIs and virtual non-contrast DLCT images can facilitate image-guided biopsy of liver lesions.

Microvascular invasion (MVI) in HCC is an important risk factor leading to high recurrence rates after radical tumor resection [16]. Preoperatively constructing an effective predictive model of MVI is highly important for determining personalized treatment strategies for HCC patients [17]. Zhu et al [18] designed a study to explore the application value of DLCT in evaluating MVI in isolated HCCs with a diameter ≤ 5 cm and in serum AFP-negative patients. The single-factor analysis results showed that the *Z*-effective during the arterial phase had the best predictive effect for MVI, with an accuracy of 0.792. A risk score was constructed by combining radiological features (including the “mosaic sign,” “crown-like enhancement,” “incomplete capsule,” and “invasion of portal vein”) and DLCT quantitative parameters. The scoring system demonstrated significant predictive value, with an AUC of 0.929. This research suggested that a scoring model combining radiological features and DLCT quantitative parameters is a promising tool for preoperatively predicting MVI.

Portal vein tumor thrombus (PVTT) is the most common form of HCC vascular invasion. The median survival time of HCC patients complicated with PVTT is significantly shorter than that of patients without PVTT [19]. Pan et al [20] reported that the iodine concentration (IC) and spectral slope during the arterial and venous peak phases of DLCT were strongly correlated between distal and proximal PVTTs and HCC primary lesions. This finding can help radiologists and clinicians identify the source of portal vein thrombosis. However, this approach has application limitations for small distal PVTTs.

## The clinical application of DLCT in pancreatic diseases

### Pancreatic ductal adenocarcinoma (PDAC)

PDAC is the predominant subtype of pancreatic cancer. It has a dismal 5-year overall survival rate (approximately 9%) and is a prevalent cause of cancer-related mortality. The current applications of DLCT for PDCA include tumor detection, staging, and assessment of resectability [21]. VMIs are computed through different energy datasets in DLCT. Typically, lower-energy VMIs can increase the CT values and tissue contrast of vessels and abnormally enhanced lesions, aiding in the detection of pancreatic lesions. Nagayama et al [22] reported that in dynamic enhanced pancreatic DLCT scans, the quality of lower-energy VMI images (40–60 keV) surpassed that of conventional mixed-energy images. Compared with conventional pancreatic parenchymal phase images, lower-energy portal venous phase imaging may yield diagnostically adequate tumor conspicuity. This enhancement holds potential significance for the early detection of tumor tissue in clinical practice. On the basis of this technical advantage, Liu et al [23] reported that quantitative parameters derived from DLCT may have the potential to preoperatively evaluate the histopathological differentiation grade of PDAC.

For locally advanced PDAC patients, neoadjuvant chemotherapy (NAC) is always recommended to improve surgical efficacy and survival rates [24]. A novel study conducted by Fujita et al [25] included 67 PDAC patients who underwent DLCT examinations before treatment and analyzed the relationship between lesion ECV and tumor NAC response. The results demonstrated that PDAC patients with a lower ECV may exhibit a better response to NAC, suggesting that DLCT-derived ECV might be a useful biomarker for predicting NAC response in PDAC patients. In addition, Fukukura et al [26] reported that the ECV fraction determined by equilibrium contrast-enhanced DLCT may predict the survival of patients with stage IV PDAC treated with chemotherapy.

### Pancreatic neuroendocrine tumors (pNETs)

pNETs account for 31.5% of all neuroendocrine tumors and 3% of all primary pancreatic tumors. Early detection and accurate localization of lesions are crucial for selecting treatment strategies [27]. Yuan et al [28] reported that the signal-to-noise ratio and contrast-to-noise ratio of 40 keV-VMIs in DLCT images were greater for pNENs than for other VMIs. Compared with conventional images, the use of IC maps and *Z*-effective maps improves objective image quality and reader preference. These findings could have important clinical implications for formulating treatment strategies.

**Fig. 2:**
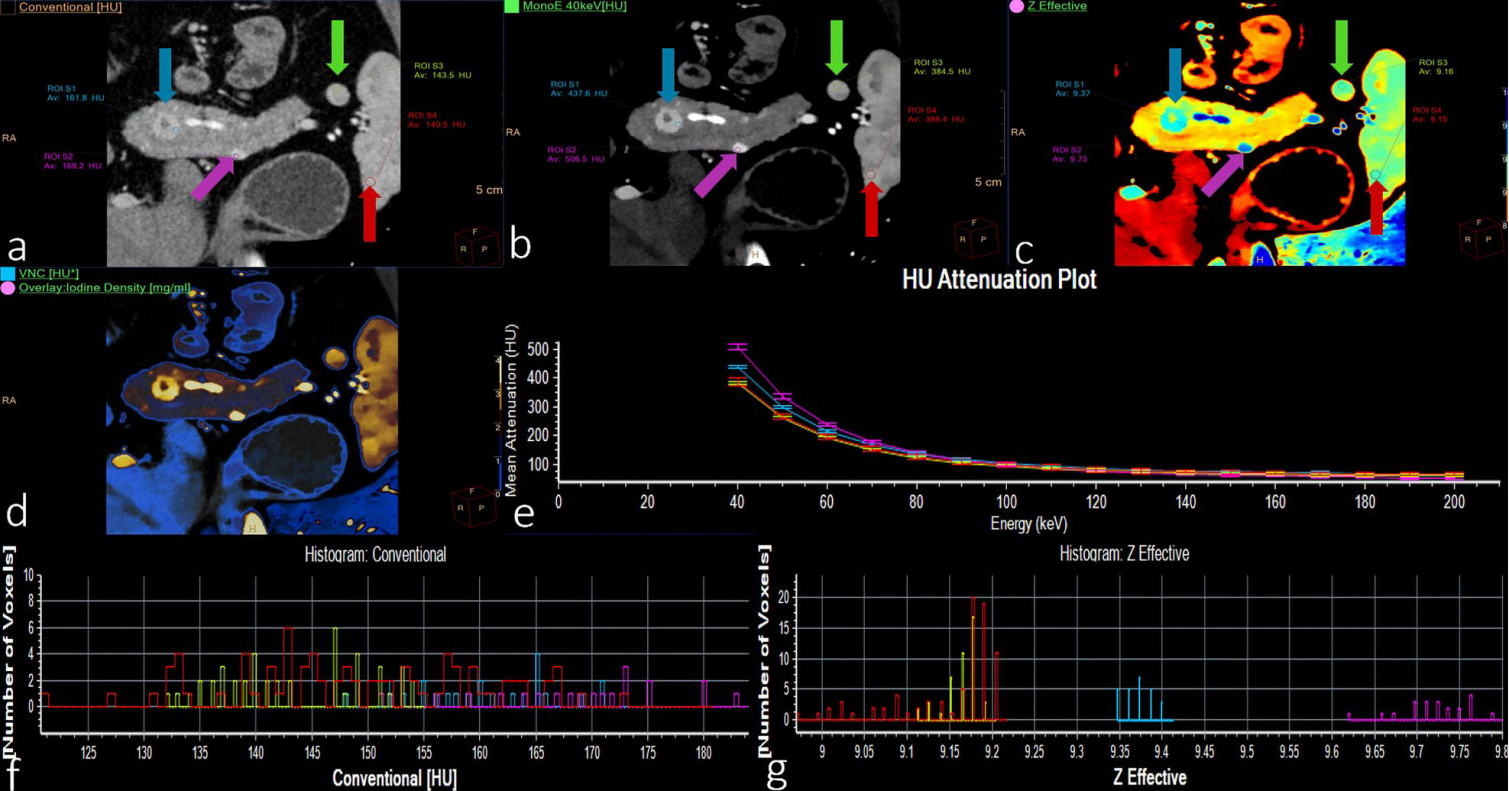
A 34-year-old male patient presented with repeated fatigue for 3 months. The pathological diagnosis was multiple islet cell tumors. There are four regions of interest, including the S1 (tumor, blue arrow), S2 (tumor, purple arrow), S3 (accessory spleen, green arrow), and S4 (spleen, red arrow) lesions. Arterial phase CT and postprocessing images: **a** conventional images; **b** 40 keV images; **c** *Z*-effective map; **d** fusion map of iodine density with virtual plain scan; **e** energy decay diagram; **f** histogram of conventional CT values; and (**g**) histogram of effective atomic order values. **b**–**d** Demonstrate the 40 keV CT values, and the *Z*-effective and VNC CT values of S1 and S2 are different from those of S3 and S4. The energy decay curves of S3 and S4 completely coincide, which are different from those of S1 and S2 in **e**. **f** Shows that the histograms of the conventional CT values of S1–S4 basically coincide. A histogram of the effective atomic order values is shown in **g**, where S3 and S4 are different from S1 and S2

Well-differentiated NETs and poorly differentiated neuroendocrine carcinomas (NECs) are two types of tumors with different clinical manifestations, histopathological characteristics, genetic features, and treatments. Accurate preoperative differentiation of their histological subtypes is beneficial for making personalized treatment decisions. Wang et al [29] retrospectively analyzed the DLCT features of 104 patients with pNETs (including 89 with well-differentiated NETs and 15 with NECs). The results showed that iodine density and the *Z*-effective in the portal venous phase were independent predictors of NEC (with AUCs of 0.897 and 0.884, respectively). The predictive accuracy of combining these two quantitative parameters was 0.921, suggesting the important clinical significance of DLCT quantitative parameters in identifying high-risk subtypes of pNETs. Figure 2 illustrates the DLCT features of pancreatic islet cell tumors.

## The clinical application of DLCT in gastrointestinal diseases and acute abdominal conditions

### Gastric cancer

CT plays a pivotal role in the early detection, tumor staging, prognosis prediction, and treatment efficacy assessment of gastric cancer. Liu et al [30] evaluated the optimal keV for visualizing gastric cancer and investigated its value in depicting lesions and identifying depth invasion via VMIs on DLCT. They found that the VMI at 40 keV demonstrated the highest performance, as evidenced by both objective metrics and subjective assessments of gastric cancer. This led to enhanced lesion visualization and improved accuracy in determining the T stage. Lymph node metastasis in gastric cancer patients is an important factor affecting patient prognosis. Conventional CT has certain limitations in distinguishing lymph node metastasis in gastric cancer patients. A prospective study [31] enrolled fifty-five gastric cancer patients, with 267 successfully matched lymph nodes (90 metastatic, 177 nonmetastatic) that were labeled on preoperative DLCT. The results showed that quantitative parameters derived from DLCT (arterial phase CT attenuation on 70 keV images, venous phase electron density, and clustered features) exhibited superior diagnostic efficacy for preoperatively identifying lymph node metastases in gastric cancer patients compared to traditional single-energy CT. This enhanced accuracy contributes to improved clinical staging of the N stage and may prompt surgeons to pay more attention to the suspected lymph node.

**Fig. 3:**
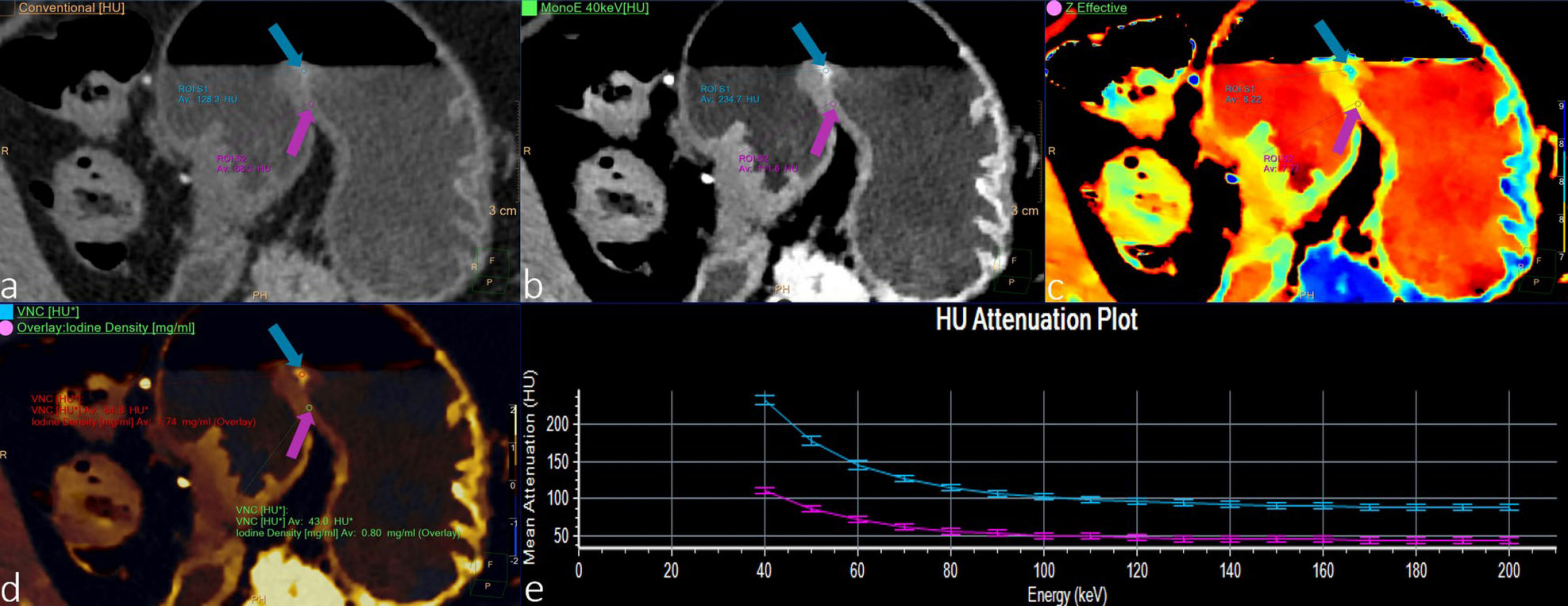
A 67-year-old female patient presented with recurrent abdominal pain, bloating, and acid reflux for six months. The pathological diagnosis was intramucosal carcinoma of the gastric horn. Arterial phase CT and postprocessing images: **a** conventional image; **b** 40 keV images; **c** *Z*-effective map; **d** fusion map of iodine density with virtual plain scan; and (**e**) energy decay diagram. Lesion S1 (blue arrow) was the tumor, and lesion S2 (purple arrow) was the surrounding normal gastric wall. **a** Shows a suspicious thickening of the gastric angular mucosa. **b**–**d** Clearly show the tumor boundary and parameter abnormalities. **e** Shows that the contrast of the two images is the highest at 40 keV, and the contrast gradually decreases with increasing energy level

Moreover, DLCT facilitated the prediction of microsatellite instability (MSI) status and Ki-67 expression in gastric cancer. Zhu et al [32] constructed a combined model using DLCT parameters and demonstrated its efficacy in the preoperative prediction of MSI. Moreover, the results indicated that the combined model may be an efficient tool for tumor recurrence risk stratification after surgery. Such a model holds promise for clinicians in selecting optimal treatment approaches to mitigate tumor recurrence risk and forecast clinical prognosis in patients with gastric cancer. Mao et al [33] reported a positive correlation between iodine density and Ki-67 expression in gastric cancer and a negative correlation between electron cloud density and Ki-67 expression, both of which can be used to accurately evaluate the Ki-67 expression status. The ability of spectral CT to predict the pathologic response after NAC has been confirmed in locally advanced gastric cancer (LAGC) patients. Li et al [34] reported that spectral CT and diffusion-weighted imaging are equally useful imaging techniques for predicting the pathologic response to NAC in LAGC patients. The combination of a normalized IC at the delayed phase and an apparent diffusion coefficient achieved significant incremental benefits and was related to patient disease-free survival. Figure 3 illustrates the DLCT features of gastric cancer (stage T1).

### Colorectal cancer (CRC)

CRC is the fourth most deadly cancer worldwide, with almost 900,000 deaths annually. Existing studies have indicated that DLCT is an effective tool for the preoperative noninvasive assessment of the biological characteristics of CRC. Chen et al [35] revealed that the quantitative parameters of preoperative DLCT could effectively predict the pathological T stage and histological grade of CRC. The *Z*-effective, iodine density, and spectral curve slope of T3/T4 stage lesions were significantly greater than those of T1/T2 stage lesions, and the above parameters were lower in poorly differentiated tumors than in well-differentiated tumors. Additionally, Sun et al [36] indicated that the ECV based on DLCT measurements can be used for preoperative noninvasive diagnosis of colorectal adenocarcinoma pT staging with excellent diagnostic efficacy. These findings may provide a new imaging marker for the preoperative evaluation of CRC and help clinicians formulate individualized treatment earlier.

The expression of Ki-67 and metastatic lymph nodes are significant biomarkers for evaluating the prognosis of CRC patients. Chen et al [37] investigated the value of DLCT quantitative parameters for evaluating the expression of Ki-67 in CRC. The results showed that the CT values at 40 keV, the *Z*-effective, the IC, and the slope of the spectral Hounsfield unit (HU) curve in the venous phase can provide valuable information for distinguishing Ki-67 high- and low-expression states in CRC. Furthermore, DLCT also has good predictive ability for evaluating metastatic lymph nodes in CRC patients. Liu et al [38] revealed that the quantitative parameters obtained from DLCT could enhance the precision of diagnosing metastatic lymph nodes in individuals with pT1-2 rectal cancer. The best diagnostic accuracy is attained by combining Zeff with the short-axis diameter of the lymph nodes. Moreover, a recent study [39] revealed that the HU and ICs and the effective *Z* value in DLCT images decreased significantly after radiochemotherapy, which provides important information for predicting treatment efficacy.

### Inflammatory bowel disease and intestinal ischemic lesions

**Fig. 4:**
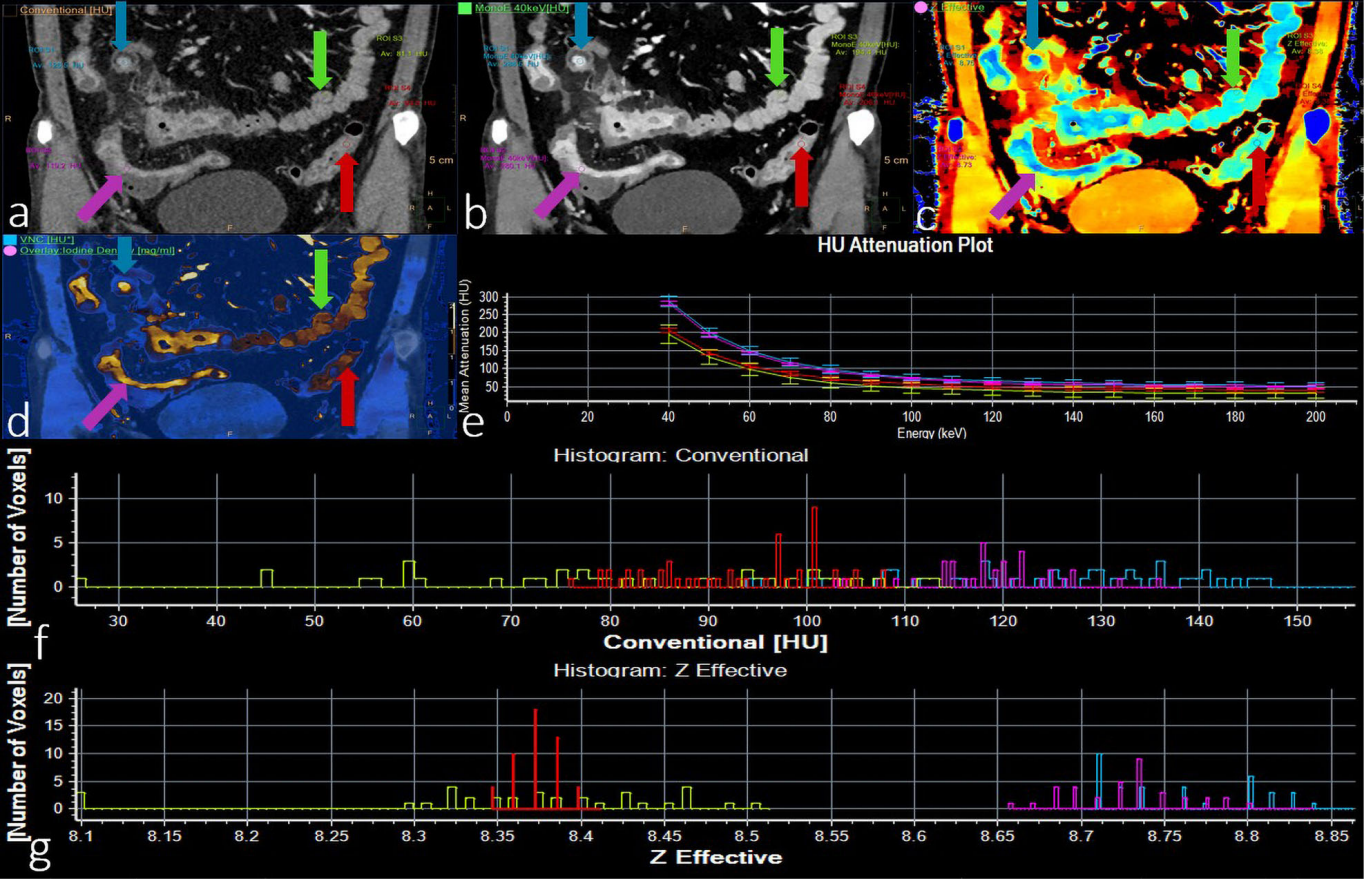
A 43-year-old male patient with CD (Montreal Type A3L3B3, CDAI score 389.55). Arterial phase CT and postprocessing images: **a** conventional images; **b** 40 keV images; **c** *Z*-effective map; **d** fusion map of iodine density with virtual plain scan; **e** energy decay diagram; **f** histogram of conventional CT values; **g** histogram of effective atomic order values. Lesions S1 (blue arrow) and S2 (purple arrow) are active CD lesions, while S3 (green arrow) and S4 (red arrow) are contralateral normal intestinal walls. **b**–**d** Show the postprocessing parameters (40 keV CT values, *Z*-effective, and VNC CT values). The above values of S1 and S2 are different from those of S3 and S4. The energy decay curves of S1 and S2 completely coincide, which are different from those of S3 and S4 in **e**. **f** Shows that the histograms of the conventional CT values of S1–S4 basically coincide. A histogram of the effective atomic order values is shown in **g**, where S1 and S2 are different from S3 and S4

Crohn’s disease (CD) is an inflammatory condition that can manifest throughout the gastrointestinal tract [40]. Huang et al [41] demonstrated that employing a combination of parameters from DLCT yields increased sensitivity and specificity for distinguishing between CD, ulcerative colitis, and intestinal tuberculosis. These findings suggest a potentially pivotal role for these strategies in treatment guidance. Taguchi et al [42] reported that DLCT assists in the quantitative assessment of CD, thereby enhancing diagnostic confidence. These advanced parametric DLCT images hold promising potential as biomarkers for the diagnosis, risk stratification, monitoring of disease progression, and outcome prediction of CD. Lee et al [43] demonstrated that low monoenergetic imaging (40 keV) provided the best contrast-to-noise ratio and the best diagnostic performance for active CD. Kim et al [44] showed that an iodine density map can also be used to monitor the activity status of CDs. Figure 4 shows the DLCT features of patients with CD (Montreal type A3L3B3, CDAI score 389.55).

DLCT may provide an effective and accurate imaging tool for evaluating the severity of acute small bowel obstruction ischemia [45]. Oda et al [46] reported a case of ischemic acute small bowel obstruction for which DLCT scanning and multiparameter postprocessing provided confidence in the diagnosis. Compared with high-energy and conventional mixed-energy images, low-energy (40 keV) DLCT images were optimal for displaying ischemic intestines. Compared with those of the normal area, the iodine density and *Z*-effective of the ischemic areas were lower.

### Acute abdominal conditions

**Fig. 5:**
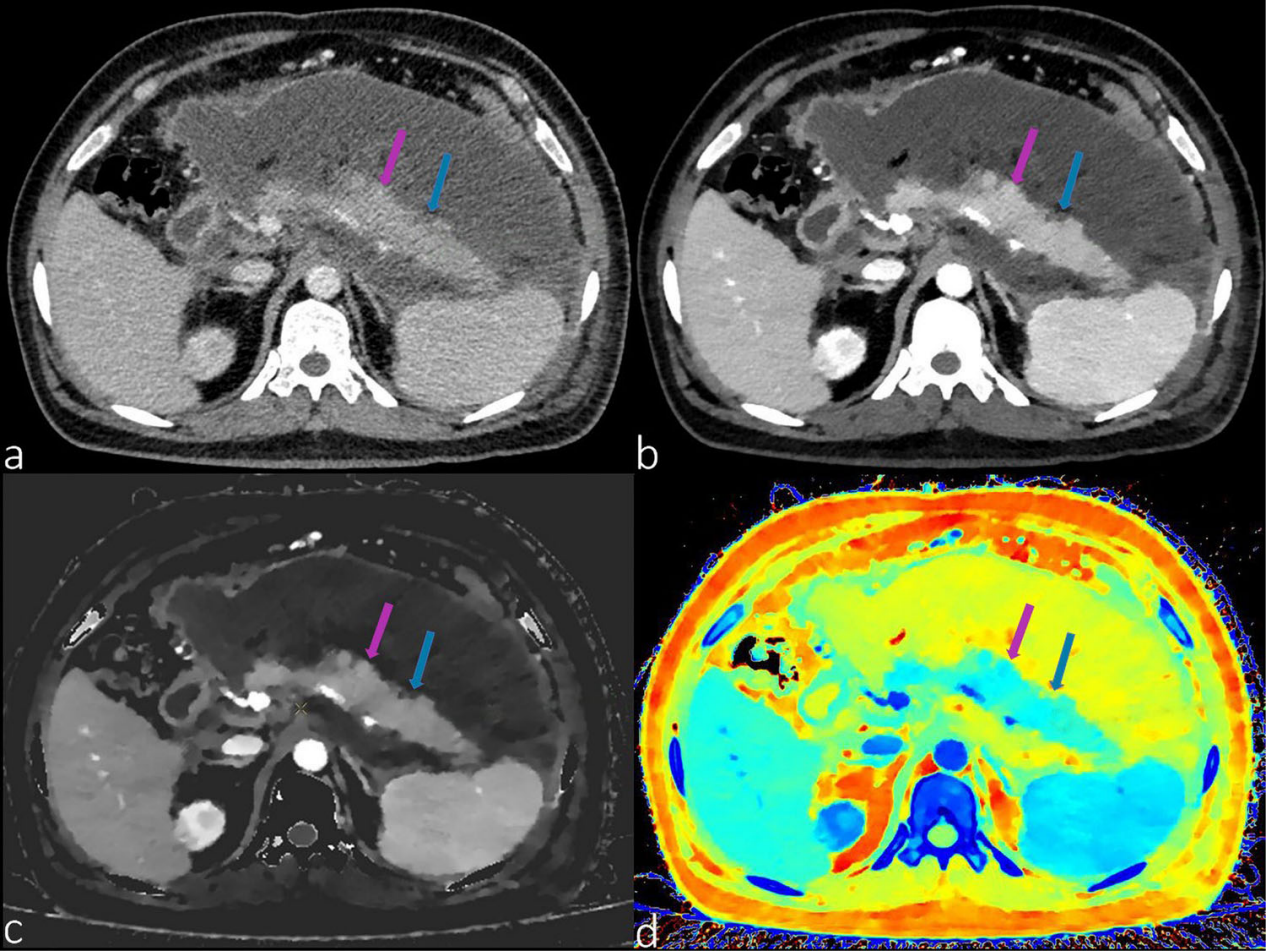
A 36-year-old male patient presented with abdominal pain for 12 days, aggravated with dyspnea for 8 days. He was diagnosed with necrotic pancreatitis. Portal phase CT and postprocessing images: **a** conventional axial images; **b** 40 keV images; **c** *Z*-effective map; and (**d**) iodine density images. The areas of residual enhancing parenchyma (purple arrow) and peripancreatic effusion (blue arrow) are better visualized in the 40 keV image and iodine density images compared to the conventional

The utility of DLCT in acute abdominal conditions, including pancreatitis, cholecystitis, and appendicitis has been revealed, particularly in enhancing lesion detectability, improving contrast resolution in inflamed tissues, and facilitating the differentiation of acute from chronic processes. Enhanced attenuation on low monoenergetic images increases bowel wall conspicuity, thereby aiding in the assessment of acutely inflamed appendices. Careful examination of these low monoenergetic images may enhance the visualization of acute inflammatory bowel conditions [47]. In addition, the low monoenergetic images provide superior visualization of pericholecystic liver enhancement and gallbladder wall integrity. These imaging characteristics may serve as potential predictors of bile culture positivity in cholecystitis patients, potentially guiding clinical decision-making regarding the necessity for intervention [48]. Similarly, low monoenergetic images enhance the assessment of inflamed pancreatic parenchyma (Fig. 5). Furthermore, these images offer superior characterization of the extent of parenchymal necrosis, facilitating more accurate classification that could improve the prediction of severe clinical outcomes [49].

## The application value of DLCT in digestive system vasculature imaging

### Superior mesenteric artery (SMA)

The SMA primarily supplies blood to the pancreas, duodenum, jejunum, ileum, and right colon. Dissection or embolism of the SMA can lead to severe consequences such as acute ischemic necrosis in the corresponding intestinal segments. In addition, rupture and hemorrhage may result in significant gastrointestinal or intra-abdominal bleeding. The development of DLCT represents a novel approach for enhancing SMA imaging quality while concurrently reducing contrast agents and radiation exposure. However, the optimal parameter settings remain empirical. Lai et al [50] proposed that the use of DLCT effectively reduces the contrast agent load while providing equivalent or superior SMA imaging quality compared to conventional contrast protocols. Notably, when the injection rate of the high-concentration contrast agent (370 mgI/mL) decreased to 2.0 mL/s, the image quality of the SMAs on 60 keV-VMIs matched that of conventional mixed energy level images. Another study [51] indicated that 40 keV and 70 keV VMIs exhibit higher signal-to-noise ratios and contrast-to-noise ratios for abdominal vessels such as the abdominal aorta and SMA. Moreover, different types of arterial plaques should be targeted with specific reconstruction energy levels. For instance, 40 keV-VMI is recommended for displaying soft plaques, while 100 keV-VMI is recommended for displaying calcified plaques.

### Portal vein

**Fig. 6:**
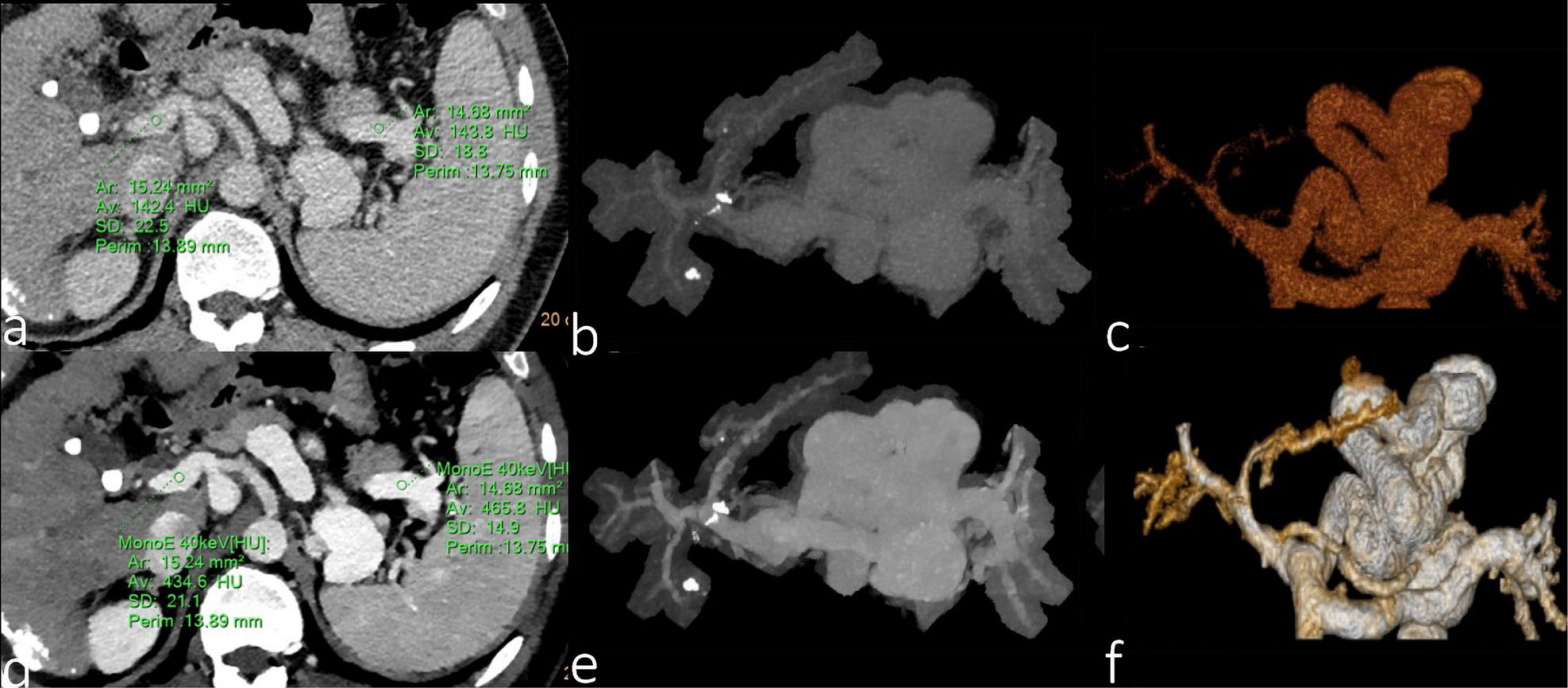
A 50-year-old male patient with hepatocellular carcinoma after interventional therapy. He had a history of cirrhosis and portal hypertension with open collateral circulation. Portal phase CT images: **a** conventional axial images; **b** conventional 3D maximum intensity projection reconstruction images; **c** conventional 3D volume rendering reconstructed images; **d** 40 keV axial image; **e** 40 keV 3D maximum intensity projection reconstruction image; and (**f**) 40 keV 3D volume rendering reconstructed images. 40 keV images including 3D reconstruction images are superior to conventional CT in displaying portal vein and collateral circulation vessels

Compared to conventional CT, DLCT has been shown to improve the quality of portal vein imaging and reduce the injected contrast volume, particularly on 40 keV virtual single energy level images, which exhibit a higher signal-to-noise ratio and contrast-to-noise ratio (Fig. 6) [52].

## Conclusions and future perspectives

The “homogeneous, simultaneous, and codirectional” spectral data acquisition mode of DLCT, coupled with its retrospective spectral image processing capabilities, has increasingly expanded its application scope in digestive system diseases, and demonstrated advantages over traditional single-energy CT, fast kVp-switching and DSCT. The primary advantage of DLCT lies in its detector-based approach, which allows for the true simultaneous acquisition of dual-energy data without the need for rapid voltage switching or dual X-ray sources. This results in reduced motion artifacts and improved temporal resolution, which is critical in abdominal imaging where peristalsis and patient movement can compromise image quality. Additionally, DLCT enables retrospective spectral analysis, which provides flexibility in cases where without the need for pre-selection of dual-energy protocols.

Specifically, DLCT can assist in the accurate quantification of hepatic fat and liver fibrosis, improve the detection rate of HCC, and assess the MVI of HCC. Additionally, it has provided additional imaging information for optimizing the preoperative CT evaluation of pancreatic tumors, predicting the efficacy of chemotherapy for pancreatic cancer, enhancing the detection of pNETs, and identifying high-risk groups. Moreover, DLCT exhibits unique advantages in the staging of gastrointestinal tumors, the evaluation of immunohistochemical characteristics, and the assessment of regional lymph node metastasis. Furthermore, in the “dual-low” scanning protocol with low contrast agents and radiation doses, DLCT can improve the imaging quality of the digestive system vasculature and enhance the ability to display vascular details. In addition, VMI combined with orthopedic metal artifact reduction algorithms for DLCT can effectively reduce artifacts caused by different types of metal implants [53], which may provide better imaging options than traditional single-energy CT for post-implantation evaluation (e.g., metal stents, coil embolization, or ^125^I radioactive seed implantation).

Currently, DLCT has demonstrated advantages in the diagnosis, prognosis, and therapeutic effect evaluation of digestive system diseases. However, in the face of a series of clinical problems that urgently need to be addressed, researchers need to delve deeper into the potential of DLCT application. In the future, more studies and evidence on DLCT, including accurate early detection of digestive system disease, guidance of the formulation of the optimal surgical method, and selection of the most beneficial population for conversion therapy, such as targeted immunity of middle and advanced digestive system tumors, are needed, as well as the identification of excellent biomarkers for predicting the efficacy of treatments for digestive system diseases. In the future, photon counting detector CT [54] with superior spectral resolution, reduced electronic noise, and enhanced image contrast, could be beneficial in the imaging of the digestive system. In addition, artificial intelligence has the potential to enhance image separation, reconstruction, and diagnostic capabilities through automation and deep learning algorithms. We believe that the combination of spectral CT with artificial intelligence will help radiologists enhance their diagnostic performance and confidence.

## Abbreviations

*Z*-effective: Effective atomic number
CRC: Colorectal cancer
CD: Crohn’s disease
DLCT: Dual-layer spectral detector CT
DSCT: Dual-source CT
ECV: Extracellular volume
IC: Iodine concentration
LAGC: Locally advanced gastric cancer
MSI: Microsatellite instability
MVI: Microvascular invasion
NAC: Neoadjuvant chemotherapy
NECs: Neuroendocrine carcinomas
PDAC: Pancreatic ductal adenocarcinoma
pNETs: Pancreatic neuroendocrine tumors
PVTT: Portal vein tumor thrombus
SMA: Superior mesenteric artery
VMIs: Virtual monoenergetic images
